# A Case of Acute Severe Ulcerative Colitis Triggered by COVID-19 Infection

**DOI:** 10.7759/cureus.34369

**Published:** 2023-01-30

**Authors:** Pankaj Nawghare, Shubham Jain, Sanjay Chandnani, Pravin Rathi

**Affiliations:** 1 Gastroenterology, Nair Hospital, Mumbai, IND

**Keywords:** management, triggers, bloody diarrhea, ulcerative colitis, covid-19

## Abstract

Ulcerative colitis is a chronic inflammatory bowel condition. One of the theories for its etiopathogenesis is gastrointestinal infections. Although COVID-19 primarily affects the respiratory tract, gastrointestinal involvement is also common. We have reported a case of a 28-year-old male who presented with bloody diarrhea, diagnosed with acute severe ulcerative colitis, triggered by COVID-19 infection after known triggers were excluded.

## Introduction

The world is currently experiencing a large-scale infection due to the SARS-CoV-2 coronavirus. The gastrointestinal system is frequently implicated, even though the respiratory system is the one it predominantly affects [1]. The pathophysiology of ulcerative colitis (UC), a chronic inflammatory illness, is a result of a complicated combination between environmental variables and genetic vulnerability. Inflammatory bowel disease (IBD) patients are not thought to have a significant risk of contracting COVID-19 infection, according to the most recent research [2]. However, patients receiving immunosuppressants have a greater risk for COVID-19-induced pneumonia [3]. Many gastrointestinal infections, including *Mycobacteria paratuberculosis, Chlamydia, Listeria monocytogens,* and many others have been proposed as causes of UC. We hereby present a case of acute severe UC (ASUC) precipitating due to COVID-19 infection.

## Case presentation

A 28-year-old male presented with a history of fever and cough for the last three days with a history of close contact with a COVID-19 confirmed case. He did not have any GI complaints during the presentation. He denied any addiction and his past history was unremarkable. He was vitally stable. He was tested with a throat swab for COVID-19 PCR, which came positive. According to the Indian Council of Medical Research (ICMR) guidelines, he was categorized as having a mild COVID-19 infection [4]. His saturation on admission was 99% and baseline investigations were also normal (Table 1).

**Table 1:** Baseline investigations of patient on admission M: Male; F: Female; MCV: Mean corpuscular volume; AST: Aspartate aminotransferase; ALT: Alanine aminotransferase; BUN: Blood urea nitrogen; CRP: C-reactive protein; ESR: Erythrocyte sedimentation rate

Parameter	Patient Value	Normal Range
Hemoglobin (g/dl)	10.3	M: 14-18; F: 12-16
Platelets (/µL)	6,98,000	1,50,000- 4,50,000
Leucocytes (/µL)	6800	4000-11000
MCV (fL)	82.4 fl	80-100
Total Protein/ Albumin (g/dL)	7.1 / 4.4	Protein: 6-7.5; Albumin: 3-4.5
T. Bilirubin/ Direct (mg/dL)	0.4 / 0.1	T. Bilirubin: 0.3-1; Direct: 0.1-0.3
AST/ALT (U/L)	10/12	5-40
Alkaline phosphate (U/L)	114	80-310
BUN/ Creatinine (mg/dL)	10 / 0.3	BUN: 5-20; Creatinine: 0.8-1.5
CRP (mg/L)	4	<6
ESR (mm/hr)	11	M: ≤ 15; F: ≤ 20
D-dimer (mcg/mL)	0.1	<0.4

**Figure 1:**
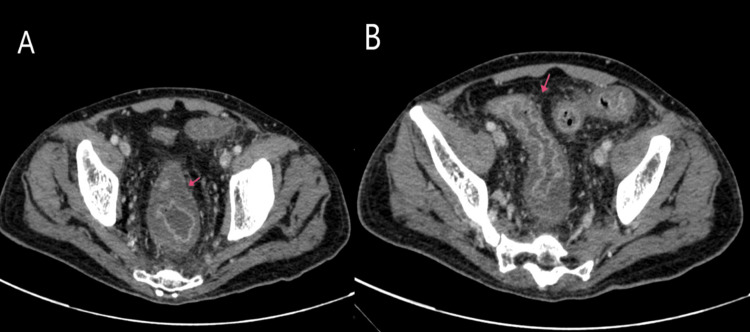
Computed Tomography (CT) showing mural thickening with peri-colic fat stranding (Red arrow) involving rectum (A) and sigmoid colon (B)

He was admitted for isolation and started on tablet hydroxychloroquine. He improved after treatment and his complaints settled down. By the fifth day of treatment, he started complaining of seven to eight episodes of bloody diarrhea associated with fever. He was febrile (temperature - 100.6 F) with a pulse rate of 124/min. There was no abdominal pain or vomiting. The rectal examination was normal, and the proctoscopy revealed red velvety rectal mucosa. However, his repeat swab of COVID-19 came negative. Due to the short history, the patient was evaluated for infective colitis. Stool examination revealed 90-100 red blood cells/HPF and 18-20 pus cells/HPF. His hemoglobin dropped from 10.3 g/dL to 8.2 g/dL during the hospital course. *Entamoeba and Clostridium difficil*e stool toxins were negative, while his CRP was >100 mg/L. His fecal calprotectin level was also elevated (2,300 µg/g). A computed tomography scan of the patient showed wall thickening involving the cecum to the rectum with vascular engorgement and surrounding peri-colic fat stranding (Figures 1A, 1B).

**Figure 2:**
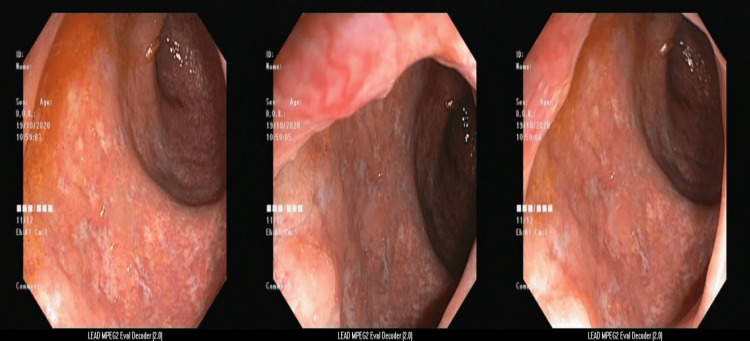
Colonoscopy suggestive of edematous, erythematous mucosa with complete loss of vascularity with multiple superficial and deep ulcerations which bleed on touch involving sigmoid colon and rectum (Mayo Grade 3 changes)

He underwent a colonoscopy, which revealed edematous, erythematous mucosa complete loss of vascularity with multiple superficial and deep ulcerations, the largest measuring 1.5 X 1 cm which bleeds on touch involving the sigmoid colon and rectum (Mayo Grade 3 changes) (Figure 2).

**Figure 3:**
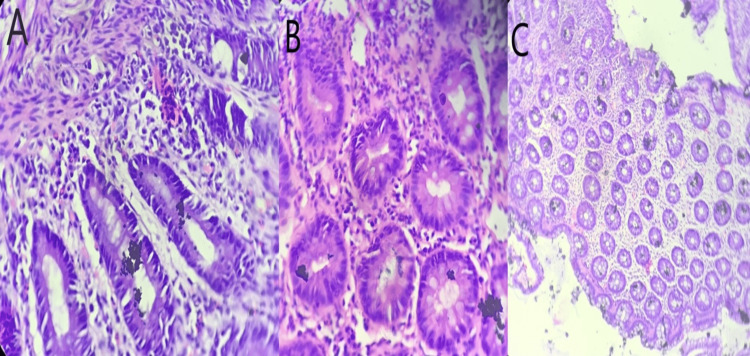
Histopathological examination showing basal plasmacytosis (A), cryptitis (B), and cryptic hyperplasia (C)

Biopsies were taken and further advancement of the colonoscope was deferred due to severe changes in the sigmoid colon and rectum. Histopathological examination of the biopsy specimen showed loss of mucin, mucosal ulcerations, basal plasmacytosis, dense mixed infiltrate with cryptitis, crypt abscesses, crypt distortion, and cryptic hyperplasia (Figures 3A-3C).

Based on clinical and histopathological findings, he was diagnosed with ASUC, probably triggered by COVID-19 infection. His Truelove-Witt’s Score (TLW) and UC Disease Activity Index (UCDAI) scores were severe and 10 respectively. He was started on intravenous steroids and he improved clinically in form of decreased stool frequency (2-3 times/day) and no blood in stool on the fifth of treatment initiation. Repeat stool examination revealed occasional red blood cells and CRP also dropped down to 7 mg/L. He was started on maintenance therapy with 5-aminosalicylic acid (5-ASA). He was discharged on oral tapering dosages of steroids with 5-ASA.

## Discussion

UC is an inflammatory gastrointestinal disorder that affects the large intestine predominantly. Studies indicate that there is an interaction of environmental factors in the genetically susceptible individual in the etiology of the disease [5]. Despite the hereditary susceptibility of UC patients, it seems that this tendency by itself is not enough to cause the illness. According to current understanding, people who are genetically predisposed develop an insufficient immune response when exposed to numerous environmental stimuli, which ultimately results in inflammatory damage to the gastrointestinal system [6].

The new coronavirus SARS-CoV-2-caused respiratory disease COVID-19, which first appeared in Wuhan, China, in December 2019, then quickly spread to the rest of the world. Although COVID-19 patients most frequently report respiratory symptoms like fever and cough, gastrointestinal symptoms like nausea/vomiting and diarrhea are also frequently noted [7]. The prevalence of GI symptoms among patients with COVID-19 ranges from various studies from 2% to 50%. Loss of appetite and diarrhea were the most commonly reported symptoms in these patients [8]. The pathophysiology of these gastrointestinal symptoms associated with COVID-19 remains unclear to date. However, SARS-CoV-2 appears to infect cells by binding to the angiotensin-converting enzyme-2 (ACE2) receptors [9]. The small and large bowels have a high expression of these ACE2 receptors [10].

In our patient, gastrointestinal manifestations developed during the COVID-19 infection. On further workups like colonoscopy and histopathological examination, findings were compatible with ASUC. Other known triggers for ASUC like infections, smoking, drugs, etc. being ruled out based on history and laboratory investigation, suggesting that the disease might be triggered by COVID-19 infection. On review of the limited literature to date, till now only a few cases have been reported in which UC was triggered by COVID-19 infection [11]. COVID-19 might unmask underlying inflammation. However, further study may be needed to investigate such occurrences. Our study's one flaw is that we didn't measure COVID-19 RNA in stool samples since stool samples are where viral RNA sheds and stays detectable for a lot longer than respiratory samples do [12].

The treatment of severe UC in people infected with COVID-19 is especially difficult. The safety of different UC treatment methods is currently being studied in the context of COVID-19. These individuals are more likely to get COVID-19 infection, allow it to replicate, and develop sepsis when given oral and intravenous corticosteroids [13]. British Society of Gastroenterology recommends the use of intravenous hydrocortisone as initial management for patients presenting with severe ulcerative with COVID-19 infection without pneumonia. However, the safety and role of steroids in COVID-19 pneumonia are still uncertain. Infliximab with continuing steroids is recommended for patients requiring rescue therapy and postponing a colectomy due to COVID-19 infection is not recommended [14].

## Conclusions

This case report highlights the importance of considering COVID-19 as a possible trigger for acute severe UC and of paying attention to its clinical features. This case highlights the need for further research into the relationship between COVID-19 and IBD, as the current understanding of this interaction is still limited.
